# Effectiveness of hypertonic saline irrigation following functional endoscopic sinus surgery: a systematic review and meta-analysis

**DOI:** 10.1016/j.bjorl.2024.101517

**Published:** 2024-11-05

**Authors:** Adriano Damasceno Lima, Rodolfo Baptista Giffoni, Julieta Arguelles-Hernandez, Gabriele Santos, Victor L.J.C. Sena, Ricardo S. Aguiar, Marcelo L.S. Cruz, Maria E.P. Dalmaschio, Marcio Nakanishi

**Affiliations:** a Universidade de Brasília (UnB), Divisão de Pós-Graduação em Ciências Médicas, Brasília, DF, Brazil; b Universidade Estadual de Feira de Santana, Departamento de Saúde, Feira de Santana, BA, Brazil; c The University of Texas MD Anderson Cancer Center, Department of Head and Neck Surgery, Houston, United States; d Centro Universitário do Espírito Santo, Colatina, ES, Brazil; e Universidade de Brasília (UnB), Hospital Universitário de Brasília (HUB), Empresa Brasileira de Serviços Hospitalares (EBSERH), Departamento de Otorrinolaringologia, Brasilia, DF, Brazil

**Keywords:** Chronic rhinosinusitis, Sinus surgery, Nasal saline solution, Post-operative, Meta-analysis

## Abstract

**Objectives:**

This study aimed to clarify the impact of hypertonic solutions on various outcomes, including persistence or reduction of nasal crusts, polypoid edema, and postoperative inflammatory symptoms.

**Methods:**

We searched PubMed, Embase, and Cochrane Central Register of Controlled Trials for studies comparing hypertonic with isotonic saline irrigation after FESS in adult patients with Chronic Rhinosinusitis (CRS). Outcomes were polypoid mucosa, nasal crusts, and variation from the baseline of Sino-Nasal Outcome Test (SNOT) 20/22 and Visual Analog Scale (VAS). For statistical analysis, we used RevMan 5.4.1, and assessed heterogeneity with I^2^ statistics.

**Results:**

We included a total of 479 patients from 7 studies. In the hypertonic saline group, there was a reduction in the nasal crust Risk Ratio (RR) (RR = 0.65; 95% CI 0.49 to 0.87; *p* = 0.004; I^2^ = 0%) after 30–45 days and severe crusts at 14–21 days (RR = 0.59; 95% CI 0.38 to 0.91; *p* = 0.02; I^2^ = 0%). Additionally, the persistence of polypoid mucosa was lower in the intervention arm (RR = 0.53; 95% CI 0.43 to 0.65; *p* < 0.00001; I^2^ = 0%) after 14–21 days. In the symptomatic evaluation hypertonic saline group showed an improvement in postoperative symptoms by a VAS Mean Difference (MD) (MD = −5; 95% CI −5.77 to −4.24; *p* < 0.00001; I^2^ = 0%) and a SNOT 20/22 Standard Mean Difference (SMD) (SMD = −1.65; 95% CI −2.7 to −0.61; *p* = 0.002; I^2^ = 93%) reduction from baseline in 30–45 days after the surgery.

**Conclusion:**

Hypertonic saline showed a superior improvement in postoperative evaluation by means of nasal crusting, mucosal healing aspect, and nasal inflammatory symptoms compared with isotonic saline irrigation.

## Highlights

• Review: hypertonic saline nasal irrigation after sinus surgery.
• Chronic rhinosinusitis: frequent inflammatory disease, may need surgery.
• Saline nasal irrigation: crucial post-surgery care for healing.
• Study: hypertonic versus isotonic saline for nasal irrigation post-surgery.
• Meta-analysis: best saline concentration for care after sinus surgery.

## Introduction

Chronic rhinosinusitis is a common inflammatory nasal disease that can significantly impair sinonasal physiology, directly contributing to the deterioration of the patient's quality of life.^1^ Although pharmacological therapy is effective for disease control, some subgroups do not respond satisfactorily to conventional clinical interventions. In these cases, Functional Endoscopic Sinus Surgery (FESS) is a beneficial alternative, aiming to restore and improve ostiomeatal ventilation and to facilitate ongoing topical medical therapy, and thus is crucial for the sustained success of long-term treatment and disease control.^2^

The success of FESS depends not only on an accurate indication and correct surgical technique but also on postoperative care to reduce inflammation and optimize early mucosa regeneration.^3^ A standardized approach to postoperative management after FESS does not exist, and current practices vary widely among surgeons.^4^ However, nasal irrigation with saline solution has become an accordant measure in early postoperative management after FESS. Studies have shown the effectiveness of this approach in flushing out inflammatory mediators, reducing nasal secretions and clotted blood, and minimizing nasal crusting and edema.^3^, ^5^, ^6^ Several solutions can be used for nasal irrigation, such as lactated Ringer's solution or corticosteroid-based solutions^7^, ^8^; however, saline solutions, based on NaCl, are the most commonly used. Saline solutions are classified as isotonic at a concentration of 0.9% and hypertonic at 2%–7%.^10^ While clinical use varies between isotonic and hypertonic, isotonic saline has generally been the preferred solution for nasal irrigation.^9^

However, recent research indicates that hypertonic saline led to a faster resolution of nasal obstruction, nasal secretion, and crusting.^10^ Despite these potential benefits, the use of hypertonic solution may be associated with irritative adverse effects,^11^ and there is no consensus on its superiority over isotonic solutions. To address the lack of concordance regarding the routine use of hypertonic nasal lavage in early postoperative management after FESS, we conducted a systematic review and meta-analysis. This study aimed to clarify the impact of hypertonic solution on various outcomes, including persistence or reduction of nasal crusts, polypoid edema, and postoperative inflammatory symptoms.

## Methods

### Eligibility criteria

The systematic review and meta-analysis were conducted following the guidelines provided by the Cochrane Collaboration and the Preferred Reporting Items for Systematic Reviews and Meta-Analysis (PRISMA) statement.^12^ Only studies that satisfied all the following criteria were included in this meta-analysis: (1) Randomized Controlled Trials (RCTs), (2) Patients with Chronic Rhinosinusitis (CRS), and (3) Trials comparing hypertonic with isotonic nasal saline irrigation (4) After FESS. Exclusion criteria were studies with (1) Other surgical modalities, (2) Patients with cystic fibrosis, (3) Nasal rinse with non-saline solutions, and (4) Use of spacers or stents.

### Search strategy and data extraction

We performed a systematic search of PubMed, Embase, and the Cochrane Central Register of Controlled Trials in October 2023 using the following terms: ‘chronic rhinosinusitis’, ‘hypertonic’, ‘sea water’, ‘isotonic’, ‘nasal’, ‘irrigation’, ‘douching’, ‘spray’, ‘sinus surgery’, ‘FESS’, and ‘postoperative’. Additionally, a manual search was carried out to discover any studies by examining the references cited in all the included studies, as well as in other systematic reviews and meta-analyses. Two authors (R.G. and G.S.) conducted the search independently. Three authors (R.G., L.S., and R.A.) independently extracted the data based on pre-defined search criteria. To extract data from images or graphs, the website WebPlotDigitizer version 4.6 (https://apps.automeris.io/wpd/) was used. The prospective meta-analysis was registered on PROSPERO on under protocol CRD42023480733.

### Endpoints and subgroup analyses

The primary outcomes of interest were the presence of polypoid mucosa appearance, nasal crusts, and variation from baseline of the Visual Analog Scale (VAS) for total nasal symptom score and Sino-Nasal Outcome Test (SNOT)-20/22. The outcomes were assessed in different time intervals across the studies, so to group the findings, we used the following time cutoffs for all outcomes: the first 7 days after surgery, 14–21 days after surgery, and 30–45 days after surgery. For studies that had assessment intervals at both 14 and 21 days, we chose to collect the earlier outcomes (14 days). We chose the polypoid edema because it is a more demarcated lesion to avoid the bias of measurement. The presence of nasal crusts was categorized as >50% or <50% crust visualized on endoscopic evaluation or according to descriptions of mild, moderate or severe crusts. We consider the accumulation of >50% of crusts and the description of severe crusts to be equivalent among the studies. All studies employed a similar framework for classifying polypoid mucosa and nasal crusts, focusing on the presence and severity of these conditions. The primary differences were in the specific terminology used and the detailed documentation of findings at different post-operative intervals. This consistency in classification allows for comparative analysis across different studies, despite the variations in assessment as shown in Table 1 of the classification of each study. Only one study reported the use of post-surgical crust debridement.^13^ However, its data were not included in the analysis of crust presence, as the results presented were collected only after 8 weeks’ post-surgery.

**Table 1:** Classification of the nasal mucosa and nasal crusts in the post-operative.

Study	Polypoid Mucosa Classification	Nasal Crusts Classification
Dawood 2022	Normal Mucosa	No Crusts
	Edematous/Cobblestoned Mucosa	Mild Crusts
	Polypoidal Mucosa	Severe Crusts
	Gross Polypoidal Mucosa	
Padiyar 2018	Normal Mucosa	No Crusts
	Edematous/Cobblestoned Mucosa	<50% Crusts
	Polypoidal Mucosa	>50% Crusts
	Gross Polypoidal Mucosa	
	Edematous/Cobblestoned Mucosa	
Tripathy 2019	Normal Mucosa	No Crusting
	Edematous/Cobblestoned Mucosa	Mild Crusting
	Polypoidal Mucosa	Severe Crusting
Vakil 2021	Normal Mucosa	No Crusts
	Edematous/Cobblestoned Mucosa	<50% Crusts
	Polypoidal Mucosa	>50% Crusts
	Gross Polypoidal Mucosa	
Low 2014.	Normal Mucosa	No Crusts
	Edematous/Cobblestoned Mucosa	<50% Crusts
	Polypoidal Mucosa	>50% Crusts
	Gross Polypoidal Mucosa	
Wang 2020.	Normal Mucosa	No Crusting
	Edematous/Cobblestoned Mucosa	Mild Crusting
	Polypoidal Mucosa	Severe Crusting
Wiikmann 2002	Normal Mucosa	No Crusts
	Edematous/Cobblestoned Mucosa	Mild Crusts
	Polypoidal Mucosa	Severe Crusts

We performed a subgroup analysis focusing on the effectiveness of the hypertonic solution in subgroups limited to cases using the high-pressure/low-volume delivery system and those using the low-pressure/high-volume delivery system, focusing on the VAS score.

### Quality assessment

Quality assessment of RCTs was performed with Cochrane’s tool for assessing bias in randomized trials.^14^ Studies are scored as having high, low, or unclear risk of bias based on five domains: selection, performance, detection, attrition, and reporting. The risk of bias assessment was conducted by two independent authors. Discrepancies between the authors were resolved through discussions, during which they presented their reasons for the disparities and reached a consensus.

### Statistical analysis

Pooled treatment effects for binary endpoints were evaluated using Risk Ratios (RR), while continuous outcomes were assessed by Mean Differences (MD) and Standardized Mean Differences (SMD), along with their corresponding 95% Confidence Intervals (95% CI). Heterogeneity was assessed using the Cochran *Q* test and I^2^ statistics. For outcomes with low heterogeneity (I^2^ < 25%), a fixed-effects model was employed. In cases of significant heterogeneity, the DerSimonian and Laird random-effects model was used. Statistical analysis was performed using RevMan 5.4.1 (Nordic Cochrane Centre, The Cochrane Collaboration, Copenhagen, Denmark). To calculate missing standard deviations, we used the RevMan online calculator (https://training.cochrane.org/resource/revman-calculator). Sensitivity analyses were conducted to evaluate the impact of individual studies on the overall results of the meta-analysis.

## Results

**Fig. 1:**
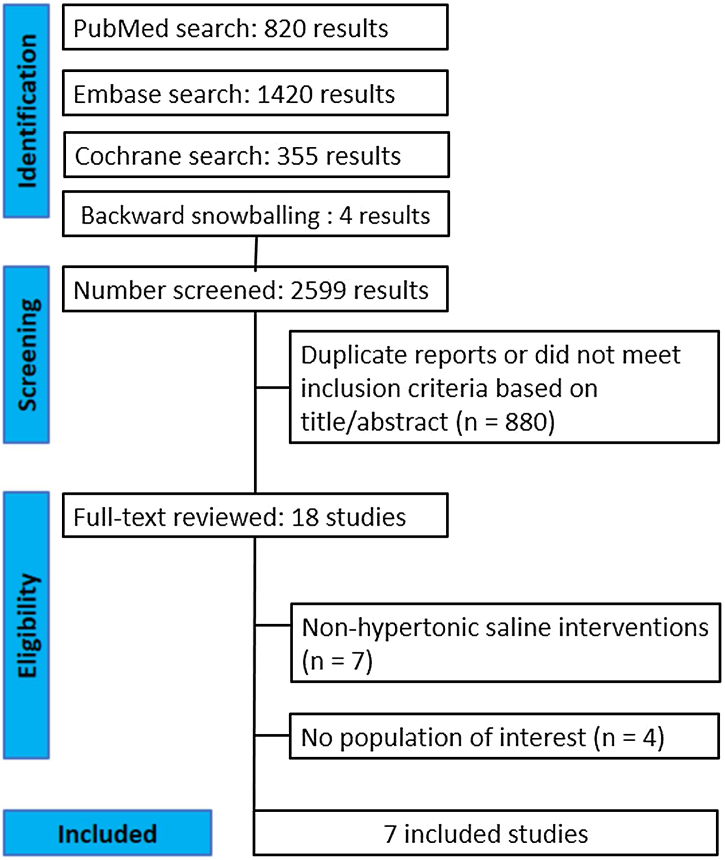
PRISMA flow diagram of study screening and selection.

The initial search generated 2599 results (Fig. 1). After elimination of duplicate records and irrelevant studies through a review of titles and abstracts, 18 records remained, each of which underwent a comprehensive review. Of these, 7 were included in the qualitative and quantitative review after the exclusion of studies with non-hypertonic saline interventions (n = 7) or no population of interest (n = 4). Ultimately, a total of 479 patients were included in this systematic review and meta-analysis.^7^, ^13^, ^15^, ^16^, ^17^, ^18^, ^19^ Postoperative irrigation with hypertonic saline solutions (2%–3%) was used in 239 patients (49.9%) with a follow-up range from 7 to 45 days after surgery. All patients included underwent FESS for CRS with or without nasal polyps, and the mean age was 37.7 years. Baseline characteristics were compared between all groups (Table 2). Table 3 provides a detailed bias evaluation of each RCT included in the meta-analysis performed using the Cochrane Risk of Bias 2 tool.^14^ Six studies were considered to have some concerns since they did not provide access to the publication protocol. One study was considered with a high risk of bias due to discrepancies in how the data are presented and the lack of a publication protocol.

**Table 2:** Baseline characteristics of included studies.

Study (first author, year)	Study design	Intervention	Delivery system	Follow-up	CRS phenotype	Age, y^a^ (hyper/iso)	Female, n (hyper/iso)	Male, n (hyper/iso)	TNP
Dawood 2022	RCT, open label	Hypertonic saline (2.3%)	High pressure/ Low volume	7, 14, and 21 days	CRSsNP	N/A	N/A	N/A	40
Low 2014	RCT, double blind	Hypertonic saline (2.7%)	Low-pressure/ High volume	7, 21, and 45 days	CRSwNP and CRSsNP	40.4/41.5	8/10	13/12	43
Padiyar 2018	RCT, open label	Hypertonic saline (3.0%)	Low-pressure/ High volume	7, 15, and 30 days	CRSwNP	34.9/34.33	10/12	20/18	60
Tripathy 2019	RCT, open label	Hypertonic saline (3.0%)	Low pressure/ High volume	7, 15, and 30 days	CRSwNP and CRSsNP	36.4/36.8	14/12	16/18	60
Vakil 2019	RCT, open label	Hypertonic saline (3.0%)	Low pressure/ High volume	7, 21, and 45 days	CRSwNP and CRSsNP	34.9/34.33	32/36	46/42	156
Wang 2020	RCT, double blind	Hypertonic saline (2.0%)	High pressure/ low volume	2, 8, 16, and 24 weeks	CRSwNP	40.78/41.80	17/16	31/29	93
Wiikmann 2002	RCT, double blind	Hypertonic saline (2.0%)	Low pressure/ LHigh volume	7 and 30 days	CRSwNP and CRSsNP	N/A	N/A	N/A	27

CRS, Chronic Rhinosinusitis; CRSsNP, Chronic Rhinosinusitis without Nasal Polyps; CRSwNP, Chronic Rhinosinusitis with Nasal Polyps; FESS, Functional Endoscopic Sinus Surgery; Hyper, Hypertonic saline; Iso, Isotonic saline; TNP, Total Number of Patients.

a Mean.

**Table 3:** Risk of bias assessment of included studies.

Study (first author, year)	Bias from radomization process	Bias due to deviations from intended interventions	Bias due to missing outcome data	Bias in menasurement of the outcomes	Bias in selection of the reported result	Overall bias
Dawood 2022	Low	Low	Low	Some concerns	Some concerns	High
Low 2014	Low	Low	Low	Low	Some concerns	Some concerns
Padiyar 2018	Low	Low	Low	Low	Some concerns	Some concerns
Tripathy 2019	Low	Low	Low	Low	Some concerns	Some concerns
Vakil 2021	Low	Low	Low	Low	Some concerns	Some concerns
Wang 2020	Low	Low	Low	Low	Some concerns	Some concerns
Wiikemann 2002	Low	Low	Low	Low	Some concerns	Some concerns

### Endoscopic outcomes

**Fig. 2:**
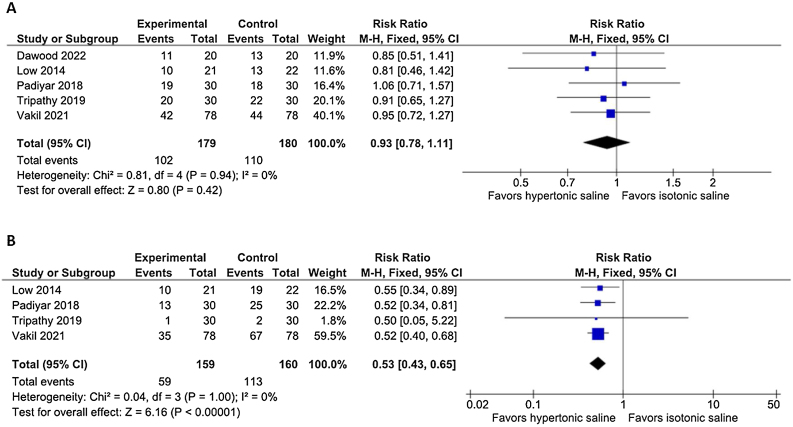
(A) The presence of crusts was similar between the hypertonic and isotonic groups after 14‒21 days (*p* = 0.42). (B) The polypoidal appearance of the mucosa showed a significant risk reduction in the hypertonic solution irrigation group at 14‒21 days (*p* < 0.00001).

**Fig. 3:**
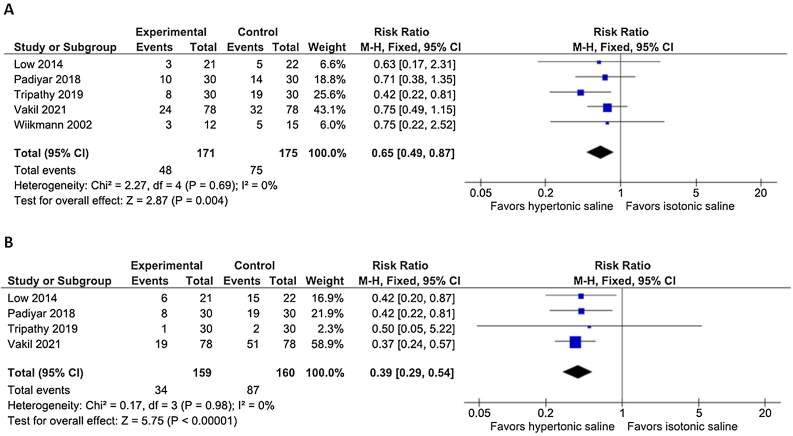
(A) The presence of crusts showed a significant risk reduction in the hypertonic solution irrigation group at 30‒45 postoperative days (*p* = 0.004). (B) The polypoidal appearance progressively improved in the hypertonic group after 30‒45 days (*p* < 0.00001).

**Fig. 4:**
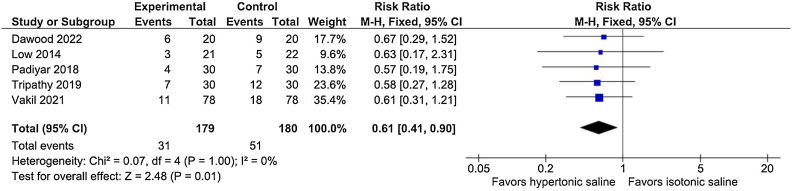
There was a significant improvement in the presence of severe crusts in the hypertonic group after 14‒21 days (*p* = 0.01).

After the first 7 days, the presence of crusts (RR = 0.99; 95% CI 0.93–1.04; *p* = 0.61; I^2^ = 0%), and the polypoid appearance of the mucosa (RR = 0.99; 95% CI 0.93–1.06; *p* = 0.80; I^2^ = 0%) were similar between the two hypertonic and isotonic groups. At 14–21 postoperative days, the presence of nasal crusts remained similar between the treatment groups (RR = 0.93; 95% CI 0.78–1.11; *p* = 0.42; I^2^ = 0%; Fig. 2A), whereas the nasal polypoid appearance was reduced in the hypertonic saline group (RR = 0.53; 95% CI 0.43 to 0.65; *p* < 0.00001; I^2^ = 0%; Fig. 2B). After 30–45 days, both the presence of nasal crusts (RR = 0.65; 95% CI 0.49 to 0.87; *p* = 0.004; I^2^ = 0%; Fig. 3A) and the polypoidal appearance of the mucosa (RR = 0.39; 95% CI 0.29 to 0.54; *p* < 0.00001; I^2^ = 0%; Fig. 3B) showed a significant risk reduction in the hypertonic group. We examined the presence of severe crust in two-time intervals. During the first 7 days of evaluation, the presence of severe crust did not significantly vary between the treatment arms (RR = 0.97; 95% CI 0.81–1.17; *p* = 0.75; I^2^ = 0%). After 14–21 days, the presence of severe crusts significantly improved in the hypertonic group (RR = 0.61; 95% CI 0.41 to 0.90; *p* = 0.01; I^2^ = 0%; Fig. 4). At 30–45 days, the presence of severe crusts maintained a progressive risk reduction in hypertonic saline intervention group (RR = 0.37; 95% CI 0.19 to 0.72; *p* = 0.004; I^2^ = 0%).

### Symptomatic outcomes

**Fig. 5:**
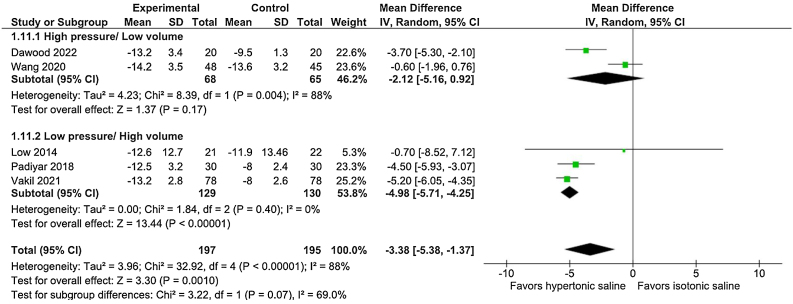
The visual analog scale for total nasal symptom score showed a significant reduction from baseline after 14‒21 days (*p* = 0.0010). The high-pressure/low-volume subgroup did not show significance; the high-volume/low-pressure group exhibited better performance by hypertonic saline in reducing the visual analog scale score.

**Fig. 6:**
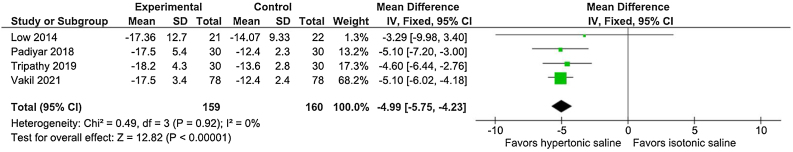
The visual analog scale for total nasal symptom score, maintaining a significant reduction from baseline after 30‒45 days (*p* < 0.00001).

**Fig. 7:**
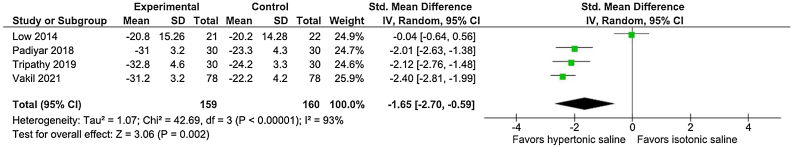
Sino-Nasal Outcome Test 20/22 after 30‒45 days, maintaining a significant difference compared to the isotonic solution group (*p* = 0.002).

After 14–21 days, VAS (MD = −3.38; 95% CI −5.38 to −1.37; *p* = 0.0010; I^2^ = 88%; Fig. 5) and SNOT-20/22 (SMD = −1.08; 95% CI −1.85 to −0.31; *p* = 0.006; I^2^ = 91%) scores showed a significantly reduction from baseline in the hypertonic group. At 30–45 postoperative days, VAS exhibited a significantly score reduction from baseline in the hypertonic group with no heterogeneity (MD = −4.99; 95% CI −5.75 to −4.23; *p* < 0.00001; I^2^ = 0%, Fig. 6). SNOT-20/22 after 30–45 days, despite maintaining a significantly reduction from baseline compared to the isotonic group, displayed high heterogeneity (SMD = −1.65; 95% CI −2.70 to −0.59; *p* = 0.002; I^2^ = 93%; Fig. 7).

### Subgroup analyses

We evaluated the effectiveness of high-pressure/low-volume and low-pressure/high-volume irrigation devices in symptomatic relief after 14–21 days of surgery. In the high-pressure/low-volume subgroup, hypertonic saline did not show significant improvement in VAS score (MD = −2.12; 95% CI −5.16 to 0.92; *p* = 0.17; I^2^ = 88%; Fig. 5). However, in the high-volume/low-pressure subgroup, hypertonic saline exhibited better performance in reducing the VAS score (MD = −4.98; 95% CI −5.71 to −4.25; *p* < 0.00001; I^2^ = 0%; Fig. 5). Importantly, the test for subgroup differences showed no significant interaction in the effect of hypertonic saline in the high-pressure/low-volume and low-pressure/high-volume irrigation subgroups (*p* = 0.07 for interaction).

## Discussion

In this systematic review and meta-analysis of 7 studies and 479 patients, we compared the efficacy of hypertonic versus isotonic saline irrigation during the early postoperative period in patients who underwent FESS for CRS. The main findings from the pooled analyses with the use of hypertonic saline were: (1) A reduction in the RR of polypoid appearance of mucosa in 47% at 14–21 days and 61% after 30–45 days and (2) A decrease in the RR of nasal crust presence in 35%, (3) A VAS reduction from baseline by −4.99 points, and (4) A reduction in the SNOT-20/22 score by −1.65 all after 30–45 days.

The functional impairment of the nasal mucosa is a natural and expected process after FESS. This phenomenon is associated with controlled trauma induced by the surgical manipulation, activating an inflammatory cascade aimed at controlling damage and restoring the mucosa to its natural state.^20^ In the initial phase, after trauma and rupture of the vessels in the mucosa, a hemostatic stage begins, with activation and formation of fibrin plugs via the coagulation process, resulting in blood crusting visible in the nose for 2–4 weeks.^11^, ^21^ In some cases, a large absence of mucosa covering exposed bone and cartilage results in an excess of crust formation, a prominent contributor to nasal obstruction, and potential infection that can compromise the healing process.^22^

Moistening the postoperative cavities contributes to decreased crusting and has favorable effects on the re-epithelialization of the mucosa.^20^, ^23^ Previous studies suggested that irrigation with hypertonic saline can reduce secretion viscosity, facilitating evacuation through mucociliary transport and physically removing crusts.^9^, ^24^, ^25^ These effects are possibly due to the hypertonic solution's ability to reduce mucus elasticity and viscosity by breaking down ionic bonds, increasing the osmotic flow between water and the layers of mucus deposited on the mucosa and improving the mucociliary function^9^, ^26^ by potentially accelerating the crusts’ dissolution. In concordance, our data suggest that hypertonic saline can reduce the risk of nasal crusts after 30–45 days compared with isotonic lavage (Fig. 3A). When we focused on individuals with severe crusts, a reduction in crust intensity was observed in the hypertonic irrigation arm after 14–21 days (Fig. 4). In the first week, there was no difference in reduction of crusts or severe crusts between the treatment groups.

Following the crust formation is an inflammatory phase that aims to clear the nasal cavity and support a mucosal transition.^21^ This stage may result in two main outcomes: a complete tissue healing with a complete epithelial metaplasia and the establishment of a new functional mucosa, or a degenerative process with an incomplete epithelium metaplasia and predominantly edema, polyps, vesicles, and granulation tissue, leading to the genesis of dysfunctional mucosa. At the beginning, these two states coexist, but depending on endogenous and environmental responses, one of the two will predominate.^21^ Our study shows that hypertonic saline irrigation results in an improvement in the polypoid appearance of the mucosa after 14–21 postoperative days (Fig. 2B), and after 30–45 days, the result is maintained (Fig. 3B) with statistical significance.^7^, ^13^, ^19^ There is no explanation for why hypertonic solution is able to reduce the polypoid appearance of the mucosa. Presumably, this effect is linked to the inhibition or modulation of certain inflammatory factors. According to some studies, hypertonic saline is believed to work by diminishing the release of interleukin-8, thereby contributing to the reduction of postoperative swelling.^25^, ^27^

We also analyzed the efficacy of hypertonic irrigation as a symptomatic reliever. This evaluation relied on two key aspects: a direct assessment of symptom severity using the VAS for total nasal symptom score and an examination of the impact of symptoms on the patient's daily activities as measured by SNOT-20/22. After 14–21 days, VAS showed a significantly reduction in the hypertonic arm (Fig. 5). Previous studies have suggested^7^ that high-volume irrigation shows superior efficacy in symptom control. In our subgroup analysis (Fig. 5), among the subgroup treated with high-volume irrigation, hypertonic saline exhibited a significant improvement over isotonic saline with a consistent similarity among the evaluated studies (I^2^ = 0%). The subgroup analysis of low-volume irrigation did not show a significant difference between hypertonic and isotonic arms and maintained heterogeneity (I^2^ = 88%; Fig. 5). However, the test for subgroup differences showed no significant interaction between the groups (*p* = 0.07 for interaction). When the treatment was extended to 30–45 days, the effects of the intervention became more pronounced and consistent across the four evaluated studies,^7^, ^16^, ^17^, ^18^ demonstrating a significant improvement compared to the baseline VAS (Fig. 6).

The SNOT is a practical tool that enables a more comprehensive assessment of CRS symptoms and their impact on the patient's quality of life.^28^ In practice, this score has proven useful for determining the disease burden and quantifying the effectiveness of clinical or surgical interventions.^29^ Our pooled analysis revealed a statistically significant improvement in SNOT after hypertonic saline lavage. At 14–21 days after surgery, SNOT maintained a statistical significance through the final measured period of 30–45 days (Fig. 7). However, despite demonstrating statistical significance, the central tendency value and confidence intervals were discreet, resulting in a lack of clinical significance. The reason for these results may lie in the structure of the SNOT questions, as few directly pertain to nasal symptoms.^29^ Thus, even with an improvement in the VAS, there may not necessarily be a direct improvement in the SNOT score.

Despite the advantages of hypertonic saline, some studies and a previous meta-analysis have documented that hypertonic saline induces a burning sensation through stimulation of nociceptive nerves, triggering the release of substance P.^30^, ^31^ Furthermore, it releases histamine, contributing to nasal hyperreactivity and hypersecretion, ultimately compromising nasal airway patency.^30^ Among the evaluated studies, only 3 reported withdrawals due to these adverse effects,^7^, ^13^, ^19^ and in most cases, losses were equivalent between the hypertonic and isotonic groups. The rest of the patients-maintained follow-up until the end of the specified duration for each study, which ranged from 21 days to 6 months. These findings suggest that hypertonic saline’s adverse effects, for the most patients, are minor and appear to be well tolerated throughout the course of treatment. The absence of double-blinding in most studies increases the likelihood of patients experiencing a placebo effect from the use of nasal solutions, especially since the double-blind studies failed to show statistically significant results in both VAS and SNOT scores, except nasal obstruction had a significant improvement using hypertonic solution comparing with isotonic one.^7^, ^13^, ^19^

### Study limitation

This study grouped different concentrations of hypertonic saline solution (2%–3%), which may compromise the quantification of the clinical effect. However, there are still no significant studies that measure and compare whether there is a real clinical difference between these saline concentrations. Additionally, most studies, despite being randomized, did not have double-blinding and did not have prolonged follow-up, restricting the evaluation of their effects only to the early postoperative period (within 30–45 days). The lack of double blinding also makes the analysis more vulnerable to measurement bias and the placebo effect, potentially skewing the perceived efficacy of the interventions under study. In the end, it is advisable to exercise caution in the interpretation of the point estimate I^2^ in meta-analyses involving a limited number of studies. In instances of smaller meta-analyses, reliance on confidence intervals is recommended to augment or substitute for the potentially biased point estimate I^2^.^32^

## Conclusion

The results of this meta-analysis, which included 479 patients, highlight that the use of hypertonic saline, compared to isotonic saline, for early management after FESS leads to better mucosal recovery and symptomatic relief. Improvement in the severity of nasal crusts and the polypoid appearance of the mucosa can be observed starting from 14 days. Similarly, hypertonic saline shows an improvement in symptoms after at least 2 weeks of use without a significant presence of side effects. These findings suggest that hypertonic solutions (2%–3%) are effective and can be safely used after endoscopic nasal surgeries.

## CRediT authorship contribution statement

A.L., R.G. and G.S. conducted a manual search to identify any additional relevant studies. A.L., R.G., L.S., and R.A. extracted the data based on predefined search criteria and performed the statistical analysis. R.G. and A.L. completed the risk of bias assessment. A.L. and R.G. led the writing of the article, with assistance from M.N., J.H. and M.D. A.L. and M.C. performed the final review of the article before submission.

## Funding

This study did not receive any funding.

## Declaration of competing interest

The authors declare no conflicts of interest.
